# Evaluating the role of pericoronary adipose tissue on coronary artery disease: insights from CCTA on risk assessment, vascular stenosis, and plaque characteristics

**DOI:** 10.3389/fcvm.2024.1451807

**Published:** 2024-10-23

**Authors:** Jingyue Wang, Huicong Zhang, Zihao Wang, Wenyun Liu, Dianbo Cao, Qian Tong

**Affiliations:** ^1^ Department of Cardiovascular Medicine, The First Hospital of Jilin University, Changchun, China; ^2^ Department of Radiology, The First Hospital of Jilin University, Changchun, China

**Keywords:** pericoronary adipose tissue (PCAT), coronary artery disease (CAD), coronary computed tomography angiography (CCTA), digital subtraction angiography (DSA), fat attenuation index (FAI)

## Abstract

**Introduction:**

Pericoronary adipose tissue (PCAT) plays a significant role in the occurrence and progression of coronary artery disease (CAD). This study investigates the relationship between PCAT and CAD, focusing on the occurrence of the disease, the severity of vascular narrowing, and the characteristics of arterial plaques.

**Methods:**

We analyzed a cohort of 152 individuals with CAD and 55 individuals with non-coronary artery disease (N-CAD). Participants underwent both coronary computed tomography angiography (CCTA) and digital subtraction angiography (DSA). Utilizing United Imaging software for artificial intelligence delineation, we measured the fat attenuation index (FAI) and volume of PCAT in the left anterior descending (LAD), left circumflex (LCX), and right coronary arteries (RCA).

**Results:**

Our findings demonstrate that while CCTA is effective in diagnosing CAD compared to DSA, its diagnostic power for individual coronary arteries remains limited. Further analysis revealed that the FAI of the RCA and the overall PCAT volume independently influenced CAD (OR: 1.057, 95% CI: 1.002 to 1.116; OR: 0.967, 95% CI: 0.936 to 0.999). FAI showed a significant independent effect on RCA stenosis (OR: 1.041, 95% CI: 1.003 to 1.081), while the fat volume of the LAD had a significant independent effect on LAD stenosis (OR: 0.884, 95% CI: 0.809 to 0.965). A higher FAI and a lower fat volume were significantly correlated with more severe vascular stenosis percentages in all three arteries (*p* < 0.05), except for the fat volume and stenosis of the LCX. Moreover, we found the significant differences in the fat volume of the LCX between different plaque types (*H* = 8.869, *p* = 0.012), with calcified plaques consistently exhibiting the lowest fat volume across all three arteries. Finally, the likelihood ratio test confirmed that incorporating the PCAT fat volume parameter of LAD significantly improved the diagnostic ability of CCTA for both CAD (*p* = 0.01543) and LAD stenosis (*p* = 0.001585).

**Conclusion:**

The quantification of PCAT has potential application value in the comprehensive assessment of CAD. It is recommended that cardiology and radiology departments consider incorporating PCAT into the assessment criteria for patients suspected of having CAD.

## Graphical Abstract

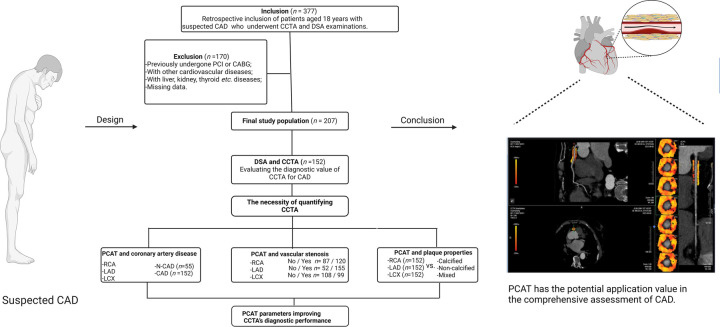

Exploring the potential application of PCAT in the assessment of CAD using CCTA. CCTA, coronary computed tomography angiography; DSA, digital subtraction angiography; LAD, left anterior descending; LCX, left circumflex artery; PCAT, pericoronary adipose tissue; RCA, right coronary artery. Created using Biorender.com.

## 1 Introduction

Coronary artery disease (CAD) is the leading cause of mortality and morbidity worldwide (1). Traditional diagnostic tools such as electrocardiography (ECG), ultrasonocardiography (UCG), and digital subtraction angiography (DSA) are the most commonly used diagnostic methods for CAD. However, these methods have respective limitations. For instance, ECG's diagnostic accuracy can vary significantly depending on the operator, and subtle ECG changes associated with early-stage CAD may not be consistently detected (2). UCG, while useful, is highly dependent on the operator's skill, and its image quality may be compromised in patients with poor echogenicity, potentially limiting its diagnostic precision. Additionally, UCG may not reliably detect small or diffuse coronary blockages that do not manifest as significant regional wall motion abnormalities (3). As for DSA, it is an invasive procedure that typically necessitates hospitalization, posing challenges for routine use in patients with suspected CAD (4). These limitations highlight the need for more advanced and comprehensive diagnostic tools that provide higher accuracy and improve ease of use (5).

In contrast to the aforementioned methods, coronary computed tomography angiography (CCTA) is less operator-dependent, offers a more streamlined measurement process, and enables automated data analysis through artificial intelligence (AI) technologies. Currently regarded as the first-line imaging modality for CAD diagnosis, CCTA boasts low radiation exposure (6) and offers excellent prognostic value (7). While DSA still offers higher diagnostic efficacy, CCTA has the advantages of being non-invasive and promoting better patient compliance (8). Crucially, CCTA allows for concurrent visualization of both the coronary arteries and pericoronary adipose tissue (PCAT), providing valuable insights into the interaction between atherosclerosis within the vessel walls and the surrounding fat deposits (9).

PCAT is the adipose tissue surrounding coronary vessels at a radial distance equal to the vessel's diameter, with a typical Hounsfield unit (HU) range of −190 to −30 (10). Research shows that PCAT, a component of epicardial adipose tissue (EAT), is strongly associated with metabolic syndrome, cardiovascular risk factors (11), and cardiovascular diseases (12). Unlike EAT, PCAT is specifically linked to atherosclerosis, as evidenced by autopsy studies (13). Previous research highlights the diagnostic potential of PCAT in CAD. Firstly, the mean attenuation or fat attenuation index (FAI) of PCAT, derived from CCTA, is emerging as a valuable non-invasive biomarker for coronary inflammation (14, 15). During vascular inflammation, suppressed adipogenesis increases lipolysis and water content in adipocytes, shifting PCAT's CT attenuation towards the aqueous phase, thereby enhancing its role in assessing coronary inflammation *in vivo* (16–18). This capability aids in early CAD risk stratification and can potentially predict risk before plaque formation (19). Secondly, a higher FAI in PCAT is associated with reduced adipocytes and lipid content (16). Studies have identified oxidized low-density lipoprotein (ox-LDL) in the plasma membranes of PCAT adipocytes, suggesting that PCAT may be a local source of ox-LDL within the coronary intima (17, 18). Since ox-LDL promotes atherosclerotic plaque progression, this association underscores PCAT's relevance in CAD risk assessment. Thirdly, an increased volume of PCAT, the fat depot closest to the coronary arteries, correlates strongly with the presence of coronary plaques (20). This correlation persists even after adjusting for cardiovascular risk factors and BMI, indicating that PCAT is more strongly associated with coronary plaque presence than other fat depots, such as epicardial or periaortic fat (20). Given that PCAT's FAI reflects both inflammation and lipid content, these findings underscore the importance of PCAT in understanding and predicting coronary atherosclerosis.

Therefore, understanding the variations in FAI and PCAT volume by CCTA could provide valuable insights for novel diagnostic and therapeutic strategies for CAD. This cross-sectional study was conducted to assess and compare independent risk factors for CAD, focusing on the extent of stenosis and the characteristics of coronary artery plaques. By analyzing these factors in relation to FAI and PCAT volume, we seek to enhance the understanding of CAD and improve its management through more precise diagnostic criteria and targeted interventions.

## 2 Methods

### 2.1 Patients and study design

**Figure 1:**
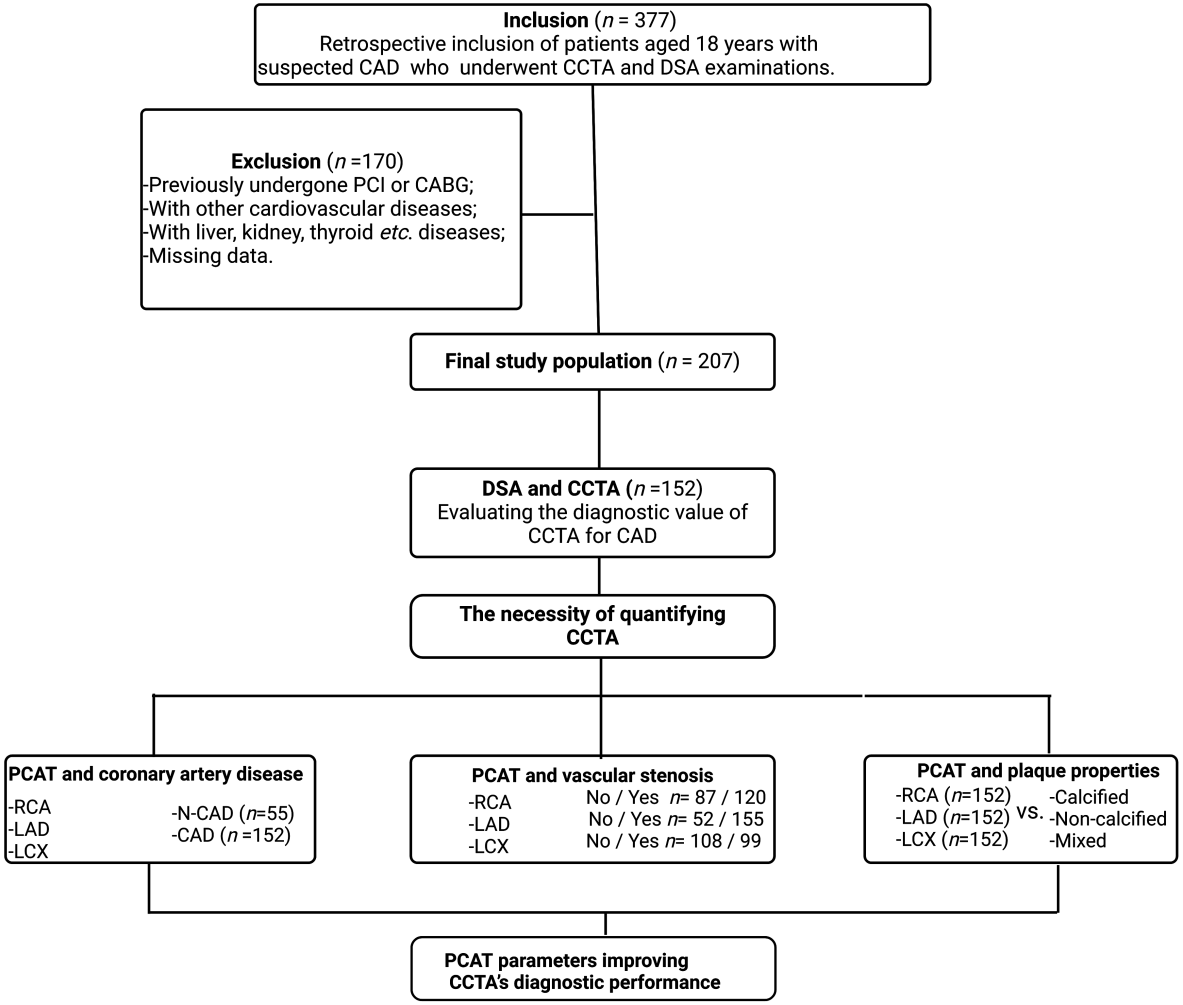
Flowchart describing the study design and selection of patients. CAD, coronary artery disease. CCTA, coronary computed tomography angiography; DSA, digital subtraction angiography; LAD, left anterior descending; LCX, left circumflex artery; PCAT, pericoronary adipose tissue; RCA, right coronary artery.

This cross-sectional study was conducted at the First Hospital of Jilin University and included patients who underwent both CCTA and DSA (Figure 1). The initial cohort comprised 377 patients, all of whom provided informed consent prior to participation.

Exclusion Criteria: After applying rigorous exclusion criteria, 207 patients were deemed eligible for inclusion in the study. Excluded patients had one or more of the following conditions: History of percutaneous coronary intervention or coronary artery bypass grafting; Myocardial infarction with non-obstructive coronary arteries; Proximal coronary artery fistulae; Severe heart valve disease; Coronary myocardial bridges; Severe hepatic or renal disease; Thyroid disorders or iodine allergy; Outpatient status or incomplete clinical data.

Patient group division: (1) Severe vs. non-severe artery stenosis: The degree of coronary artery stenosis was assessed using DSA. A 50% threshold was applied to classify stenosis as severe (≥50%) or non-severe (<50%) for the three major coronary arteries (LAD, LCX, and RCA). (2) CAD vs. non-coronary artery disease (N-CAD): Based on DSA findings, patients were retrospectively divided into two groups: CAD Group: Patients with ≥50% stenosis in at least one main coronary artery (152 patients); N-CAD Group: Patients with <50% stenosis in all three main coronary arteries (55 patients). (3) With and without stenosis: Each artery was categorized into with (narrowing percent > 0) and without (narrowing percent = 0) stenosis groups based on CCTA examination. (4) Calcified vs. non- calcified plaques: Plaque type in CAD patients was determined via CCTA. Patients were categorized as having calcified plaques (CP, HU > 150) or non-calcified plaques (NCP, HU < 150). The mixed plaque type included both calcified and non-calcified components (21).

### 2.2 DSA examination

DSA was performed following standard clinical protocols and served as the gold standard for diagnosing CAD. For the left coronary arteries (LAD and LCX), at least six projections were obtained, while a minimum of two projections were taken for the RCA. Two experienced cardiologists, each with over a decade of expertise in cardiac catheterization, independently reviewed the angiograms, blinded to the CCTA results. The measurement from the projection showing the most significant narrowing was deemed the most clinically relevant. All coronary artery segments with a diameter of ≥1.5 mm were visually and quantitatively assessed (22, 23).

### 2.3 CCTA examination

Dynamic volumetric scanning for CCTA was performed using GE Revolution 256 and Siemens Cardiac 192 systems. The scanning area extended from the tracheal ridge to 2 cm below the cardiac apex, with patients positioned supine, arms raised, and head advanced. CCTA scan parameters were standardized with a tube voltage of 100 kV and automatic current adjustment between 300 and 600 mA. The reconstruction slice thickness was set between 0.625 and 0.75 mm. An intravenous contrast agent (50–60 mL of non-ionic iodixanol) was administered at a rate of 4.5–5 mL/s, followed by 40–50 mL of physiological saline. The optimal imaging sequence for each coronary artery was selected for analysis. Two experienced radiologists, with 15 and 20 years of expertise in CCTA diagnostic imaging, independently evaluated the images. Intra- and inter-observer agreement was assessed using the intra-class correlation coefficient (ICC), with an ICC > 0.75 indicating good reliability (24).

### 2.4 PCAT parameters

**Figure 2:**
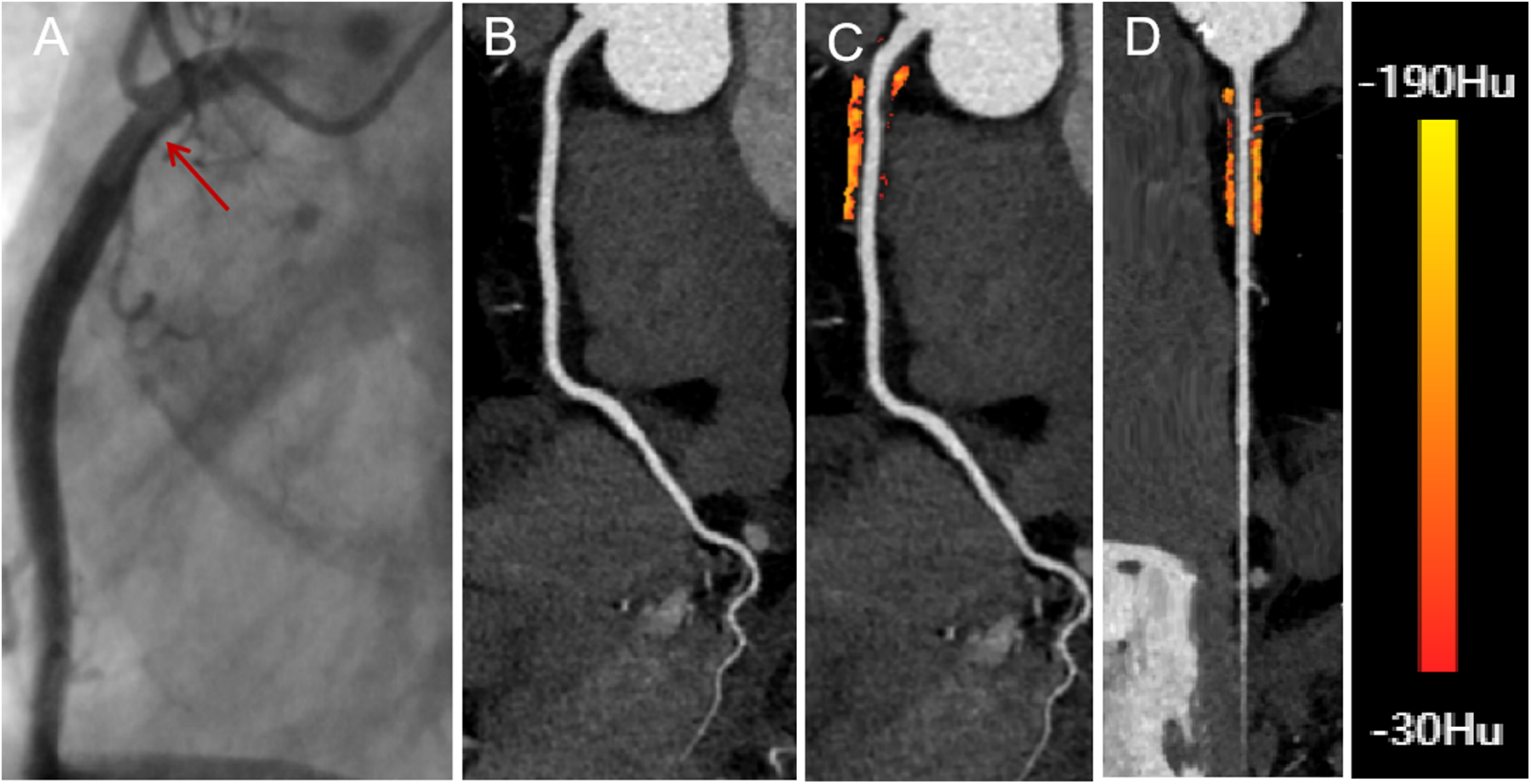
A 66-year-old male with insignificant hemodynamic stenosis in the proximal segment of the right coronary artery. **(A)** The red arrow shows a narrowing of 30% in the DSA. **(B)** CCTA with curved planar reformation of the right coronary artery shows non-calcified plaque formation in the proximal segment, with an approximate lumen narrowing of 15%. **(C)** A representative curved planar reformation image reveals a FAI [−81 HU] of PCAT, suggesting non-ischemic coronary artery narrowing. **(D)** FAI color maps of the PCAT in the proximal 10–50 mm of the right coronary artery, with a probe straightening image. PCAT color map ranges from red [−30 HU] to yellow [−190 HU]. CCTA, coronary computed tomography angiography; DSA, digital subtraction angiography; FAI, fat attenuation index; PCAT, pericoronary adipose tissue.

**Figure 3:**
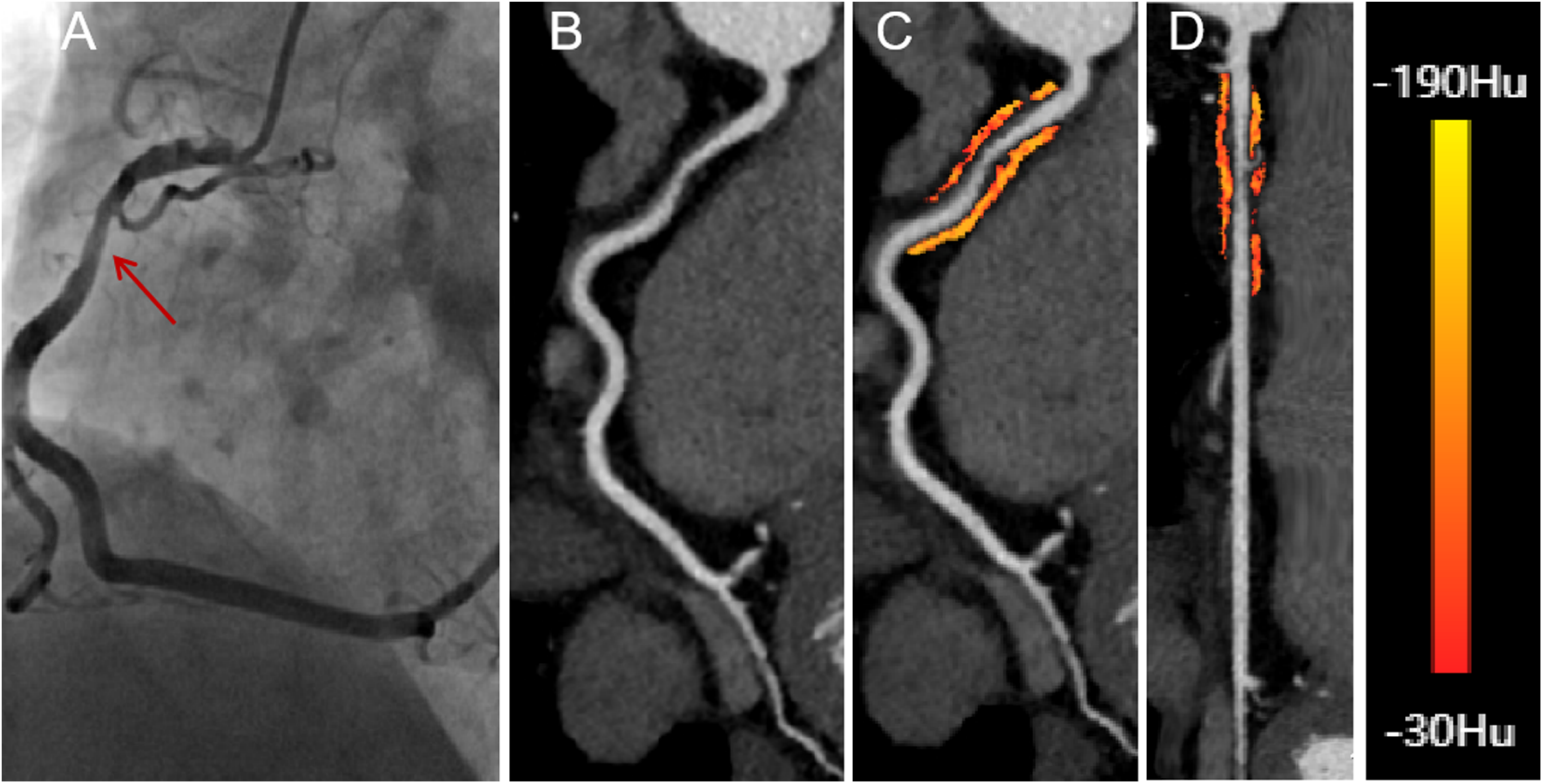
A 58-year-old male presenting with significant hemodynamic stenosis in the proximal segment of the right coronary artery. **(A)** The red arrow shows a 70% lumen narrowing in the DSA. **(B)** CCTA with curved planar reformation demonstrates non-calcified plaque formation in the proximal segment of the right coronary artery. **(C)** A representative curved planar reformation image reveals a FAI [−73 HU] of PCAT, indicative of ischemic coronary artery narrowing. **(D)** FAI color maps of the PCAT in the proximal 10-50 mm of the right coronary artery, with a probe straightening image. CT attenuation values color spectrum from red [−30 HU] to yellow [−190 HU]. CCTA, coronary computed tomography angiography; DSA, digital subtraction angiography; FAI, fat attenuation index.

CCTA images of all eligible patients were imported into United Imaging software for automated AI-based delineation of PCAT. The fat volume and FAI of PCAT within the coronary artery regions of interest were automatically analyzed using the software, following Wen's algorithm (21). PCAT was color-coded as orange or red based on attenuation values, and the AI system co-processed the imaging data to calculate the fat volume and FAI for the proximal coronary arteries. Representative AI-based delineations of fat surrounding the RCA in patients with both insignificant and significant hemodynamic stenosis are shown in Figures 2, 3.

The PCAT volume and FAI were calculated for the proximal 10–50 mm of the RCA and the proximal 0–40 mm of the LAD and LCX arteries. For the RCA, the first 10 mm of PCAT was excluded to minimize partial volume effects and artifacts caused by intraluminal contrast (25). In cases where the RCA length was less than 50 mm, the proximal limit of PCAT measurement was adjusted to within 0–10 mm of the RCA orifice. Coronary branches of the RCA and adjacent myocardial tissue were excluded from the automated PCAT analysis to avoid confounding factors (24).

### 2.5 Artery plaque properties

CCTA data were also utilized to evaluate the characteristics of proximal coronary plaques. Plaques were classified as either CP or NCP. CP refers to lesions composed of structures with CT attenuation higher than that of the coronary lumen in at least two planes with contrast enhancement. NCP is defined as lesions that can be clearly attributed to the vessel wall (in at least two views) with attenuation lower than that of the contrast-enhanced lumen. A simple threshold of 150 Hounsfield units (HU) was used to distinguish between calcified and non-calcified plaques (26). The plaque properties (CP, NCP, or mixed) for each artery were determined for patients diagnosed with CAD.

### 2.6 Baseline characteristics

Baseline characteristics were collected for all patients before DSA or CCTA examinations and included demographic and lifestyle factors (age, gender, smoking, drinking), cardiometabolic risk factors (body mass index-BMI, hypertension, diabetes, dyslipidemia, arrhythmia, fasting blood glucose-FBG, triglycerides-TG, low-density lipoprotein cholesterol-LDL-c, total cholesterol, uric acid, serum potassium-K^+^, and creatinine), as well as biomarkers of cardiovascular function and inflammation (brain natriuretic peptide-BNP, D-dimer, and white blood cell-WBC).

### 2.7 Statistical analysis

#### 2.7.1 ROC analysis

The roc function from the pROC package in R was used to construct receiver operating characteristic (ROC) curves and calculate the area under the curve (AUC) along with the corresponding confidence intervals for the logistic regression models. ROC analysis was performed to predict CAD vs. N-CAD and to assess severe vs. non-severe stenosis for each artery based on DSA examinations.

#### 2.7.2 Contingency table analysis

DSA was used as the gold standard for determining severe vs. non-severe artery stenosis. The continuous narrowing percentage obtained from the CCTA examination was also used to evaluate arterial stenosis at a threshold of 50% and compared to DSA results. Sensitivity, specificity, and Youden's index were derived from contingency tables based on the results of DSA and CCTA, which were used to assess the diagnostic performance of CCTA in CAD patients.

#### 2.7.3 Univariable association analysis

Association analyses were conducted between baseline characteristics and PCAT parameters with CAD/N-CAD and artery stenosis levels (severe or non-severe). Independent samples *t*-tests or *z*-tests were used for normally distributed quantitative variables with equal variances, while the Mann-Whitney *U* test was employed for non-normally distributed or unequal variance data. The Shapiro-Wilk test was used to assess the normality of distributions. For categorical variables, the chi-square test or Fisher's exact test was applied, depending on sample sizes and expected frequencies. The statistical significance is determined at a significance threshold of 0.05.

#### 2.7.4 Multivariate logistic regression analysis

Variables with a *p*-value < 0.05 in univariate analysis were included to identify independent effects unless otherwise stated. Odds ratios (OR) were calculated to quantify the strength of associations. The statistical significance is determined at a significance threshold of 0.05.

#### 2.7.5 Spearman correlation analysis

Spearman correlation was used to assess the relationship between PCAT parameters and stenosis of individual coronary arteries (RCA, LAD, and LCX). The statistical significance is determined at a significance threshold of 0.05. The Spearman correlation coefficient, denoted by *ρ*, indicates the strength and direction of the association between two variables (27).

#### 2.7.6 ANOVA

Analysis of variance (ANOVA) was used to evaluate differences in FAI among plaque properties (calcified, non-calcified, or mixed) for data with normal distribution and equal variances. For fat volume data that did not follow a normal distribution or had unequal variances, the Kruskal-Wallis H test was applied. The statistical significance is determined at a significance threshold of 0.05.

#### 2.7.7 Likelihood ratio test

The anova function in R was used to perform the likelihood ratio test (LRT) by comparing two nested logistic regression models: a full model and a reduced model. The full model included variables with *p* < 0.1 from the initial multivariate logistic regression, which comprised both the narrowing percentage and PCAT parameters of the arteries. The reduced model included only the narrowing percentage variables. The significance of the LRT was determined at a significance level of 0.05.

#### 2.7.8 Software

All statistical analyses were performed using SPSS version 26.0 (IBM Corp., Chicago, IL, USA), GraphPad Prism version 10.1.2 (GraphPad Software Inc., USA) and R version 4.3.1. Statistical methods were verified for accuracy by the Clinical Epidemiology Laboratory of the First Hospital of Jilin University. A *p*-value < 0.05 was considered statistically significant (28, 29).

## 3 Results

### 3.1 Evaluation of CCTA efficacy in diagnosing CAD

Both DSA and CCTA were performed on all patients to diagnose CAD. Arterial stenosis was classified as severe or non-severe based on a 50% narrowing threshold for the three major coronary arteries: the LAD, LCX, and RCA. Patients were diagnosed with CAD if stenosis ≥50% was detected in any of the three arteries on DSA; patients were classified as N-CAD if all three arteries had <50% stenosis.

**Figure 4:**
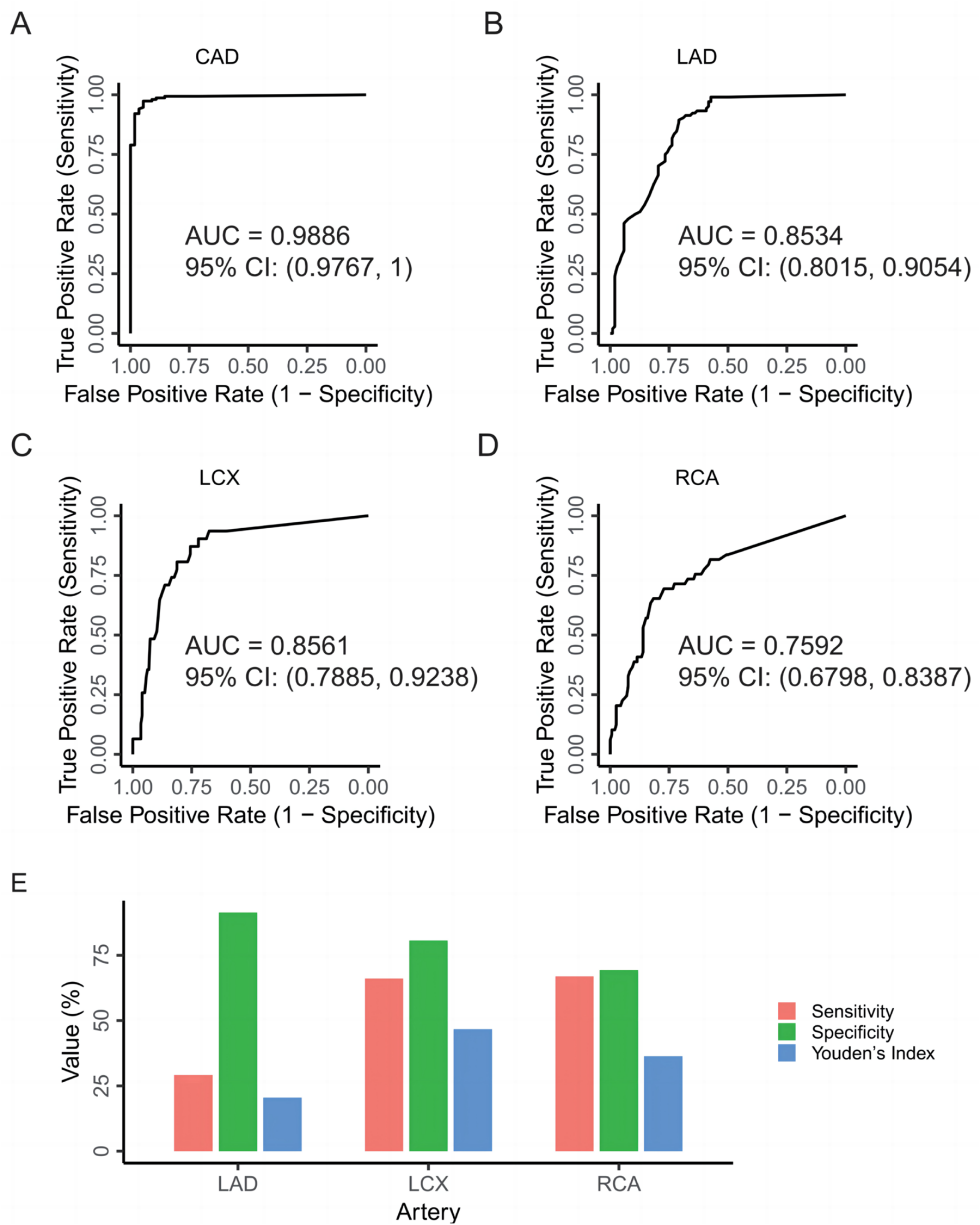
Evaluation of CCTA's efficacy in diagnosing CAD. **(A)** ROC curve for predicting CAD in DSA examinations using the narrowing percentages of the three major coronary arteries (LAD, LCX, RCA) from CCTA as predictors. **(B)** ROC curve for predicting severe vs. non-severe stenosis of the LAD in DSA examinations, with the narrowing percentage of the LAD from CCTA as the predictor. **(C)** ROC curve for predicting severe vs. non-severe stenosis of the LCX in DSA examinations, with the narrowing percentage of the LCX from CCTA as the predictor. **(D)** ROC curve for predicting severe vs. non-severe stenosis of the RCA in DSA examinations, with the narrowing percentage of the RCA from CCTA as the predictor. **(E)** Evaluating the diagnostic performance of CCTA for each vessel type compared to the DSA as the standard in CAD patients. CAD, coronary artery disease; CCTA, coronary computed tomography angiography; LAD, left anterior descending artery; LCX, left circumflex artery; RCA, right coronary artery.

Using DSA as the gold standard, we evaluated the performance of CCTA-derived narrowing percentages to predict CAD and N-CAD status. The analysis yielded an AUC of 0.9886 (95% CI: 0.9767, 1), indicating the overall efficacy of CCTA in diagnosing CAD (Figure 4A). Next, we assessed the predictive performance of CCTA for severe vs. non-severe stenosis in each of the three arteries, as determined by DSA. The AUC values were 0.8534 (95% CI: 0.8015, 0.9054) for LAD, 0.8561 (95% CI: 0.7885, 0.9238) for LCX, and 0.7592 (95% CI: 0.6798, 0.8387) for RCA, demonstrating good performance but with room for improvement (Figures 4B–D).

We then evaluated the performance of CCTA in predicting severe vs. non-severe stenosis in CAD patients specifically, using the same 50% stenosis threshold applied in DSA. A contingency table was constructed to compare the diagnostic outcomes of CCTA and DSA (Table 1). For the LAD, the sensitivity, specificity, and Youden's Index of CCTA were 91.35%, 29.17%, and 20.52%, respectively. For the LCX, these values were 80.65%, 66.12%, and 46.77%. For the RCA, sensitivity, specificity, and Youden's Index were 69.39%, 66.99%, and 36.38%, respectively (Figure 4E). These results suggest that while CCTA shows diagnostic value in detecting CAD, its performance when relying solely on stenosis measurements could be enhanced by incorporating additional parameters, such as PCAT metrics.

**Table 1:** Comparison of assessing vascular stenosis of LAD, LCX, and RCA in patients with coronary artery disease by CCTA and DSA.

CCTA (LAD)	DSA (LAD)		Sum (*n*)
	<50% (*n*)	≥50% (*n*)	
<50% (*n*)	14	9	23
≥50% (*n*)	34	95	129
Sum (*n*)	48	104	152
CCTA (LCX)	DSA (LCX)		Sum (*n*)
	<50% (*n*)	≥50% (*n*)	
<50% (*n*)	80	6	86
≥50% (*n*)	41	25	66
Sum (*n*)	121	31	152
CCTA (RCA)	DSA (RCA)		Sum (*n*)
	<50% (*n*)	≥50% (*n*)	
<50% (*n*)	69	15	84
≥50% (*n*)	34	34	68
Sum (*n*)	103	49	152

LAD, left anterior descending artery; LCX, left circumflex artery; RCA, right coronary artery.

### 3.2 Validation of PCAT’s diagnostic value for CAD

PCAT has the potential to enhance the diagnostic performance of CCTA. The ICCs for intra-observer and inter-observer agreement were 0.88 and 0.82, respectively, indicating good reproducibility. Two key PCAT parameters—FAI and volume—were selected to assess their impact on CCTA's diagnostic accuracy. These parameters were calculated for the three major coronary arteries: the RCA, LAD, and LCX.

In addition to PCAT parameters, several demographic and lifestyle factors (age, gender, smoking, drinking), cardiometabolic risk factors (BMI, hypertension, diabetes, dyslipidemia, arrhythmia, FBG, TG, LDL-c, total cholesterol, uric acid, K^+^, and creatinine), as well as biomarkers of cardiovascular function and inflammation (BNP, D-dimer, and WBC), were also incorporated into the analysis. Univariate association analysis was performed to examine the relationships between these factors and CAD (see Methods). The results indicated that age, hypertension, diabetes, dyslipidemia, TG, WBC, uric acid, FBG, BNP, D-dimer, FAI of the RCA, LAD, and LCX, and overall PCAT volume were significantly associated with CAD (*p* < 0.05; Table 2).

**Table 2:** Comparison of the characteristics of patients in the coronary artery disease group and the non-coronary artery disease group.

Variables	N-CAD (*n* = 55)	CAD (*n* = 152)	Univariate		Multivariate	
			*t*/*z*/*χ^2^*	* p*-value	*β*	*p*-value
Age (y)	55.47 ± 10.46	61.47 ± 10.39	3.660	<0.001*	0.048	0.033*
BMI (kg/m^2^)	24.88 ± 3.43	25.89 ± 3.46	1.858	0.065		
Gender (M/F)	28/27	92/60	1.533	0.216		
Smoking (N/Y)	42/13	105/47	1.041	0.308		
Drinking (N/Y)	43/12	117/35	0.034	0.855		
Hypertension (N/Y)	45/10	58/94	30.795	<0.001*	1.734	<0.001*
Diabetes (N/Y)	52/3	106/46	13.757	<0.001*	0.510	0.537
Dyslipidemia (N/Y)	30/25	59/93	4.077	0.043*	0.127	0.813
Arrhythmia (N/Y)	45/10	106/46	2.987	0.084		
TG (mmol/L)	1.35 ± 0.72	1.98 ± 1.35	4.159	<0.001*	0.718	0.061
LDL-c (mmol/L)	2.78 ± 0.78	2.85 ± 0.86	0.554	0.580		
Cholesterol (mmol/L)	4.66 ± 0.89	4.57 ± 1.22	0.626	0.533		
WBC (×10^9^/L)	6.40 ± 2.05	7.26 ± 2.15	3.008	0.003*	0.194	0.106
Uric acid (μmol/L)	331.75 ± 100.44	355.21 ± 89.82	2.069	0.039*	0.001	0.823
Serum K ^+^ (mmol/L)	4.01 ± 0.26	4.02 ± 0.39	0.138	0.890		
FBG (mmol/L)	5.40 ± 0.94	6.45 ± 2.10	3.730	< 0.001*	0.361	0.183
BNP (ng/mL)						
≤100	52 (94.55%)	119 (78.29%)	7.429	0.006*	1.613	0.054
>100	3 (5.45%)	33 (21.71%)				
D-dimer (ng/mL)						
≤600	52 (94.55%)	126 (82.89%)	4.231	0.040*	1.636	0.060
>600	3 (5.45%)	25 (16.45%)				
Creatinine (μmol/L)						
≤97	51 (92.73%)	139 (91.45%)	0.000	0.992		
<97	4 (7.27%)	13 (8.55%)				
FAI (RCA) (HU)	−86.67 ± 9.62	−81.74 ± 9.24	3.952	< 0.001*	0.056	0.044*
FAI (LAD) (HU)	−82.31 ± 8.13	−80.54 ± 7.46	2.045	0.041*	−0.049	0.201
FAI (LCX) (HU)	−78.73 ± 8.25	−74.91 ± 7.90	3.036	0.003*	0.033	0.372
PCAT overall volume (mm^3^)	3,536.52 ± 1,252.87	2,929.53 ± 1,268.37	3.512	< 0.001	−0.034	0.041*

BMI, body mass index; BNP, brain natriuretic peptide; FBG, fasting blood glucose; FAI, fat attenuation index; LAD, left anterior descending artery; LCX, left circumflex artery; PCAT, pericoronary adipose tissue; RCA, right coronary artery; TG, triacylglycerol; WBC, white blood cell; M/F, male/female; N/Y, no/yes.

* *p* < 0.05.

To further explore the independent effects of PCAT parameters, multivariate logistic regression was conducted, incorporating the significant factors identified in the univariate analysis. The results showed that both the FAI of the RCA and the overall PCAT volume remained significant predictors of CAD after adjusting for other variables (Table 2). Specifically, higher PCAT volume was associated with a reduced risk of CAD (OR: 0.967, 95% CI: 0.936 to 0.999), while a higher FAI value of the RCA was linked to an increased risk of CAD (OR: 1.057, 95% CI: 1.002 to 1.116). In conclusion, both univariate and multivariate analyses support the use of FAI and PCAT volume as valuable adjuncts in CAD assessment, improving the diagnostic capabilities of CCTA.

### 3.3 Association between PCAT and artery stenosis

Given that both FAI and PCAT volume are significantly associated with CAD, and that these relationships vary across different arteries, we next examined the association between PCAT parameters and stenosis in the RCA, LAD, and LCX. Each artery was categorized into with (narrowing percent > 0) and without (narrowing percent = 0) stenosis groups based on CCTA examination (see Methods). Univariate and multivariate association analyses were then performed to assess the relationship between PCAT parameters and artery stenosis for the RCA, LAD, and LCX.

**Figure 5:**
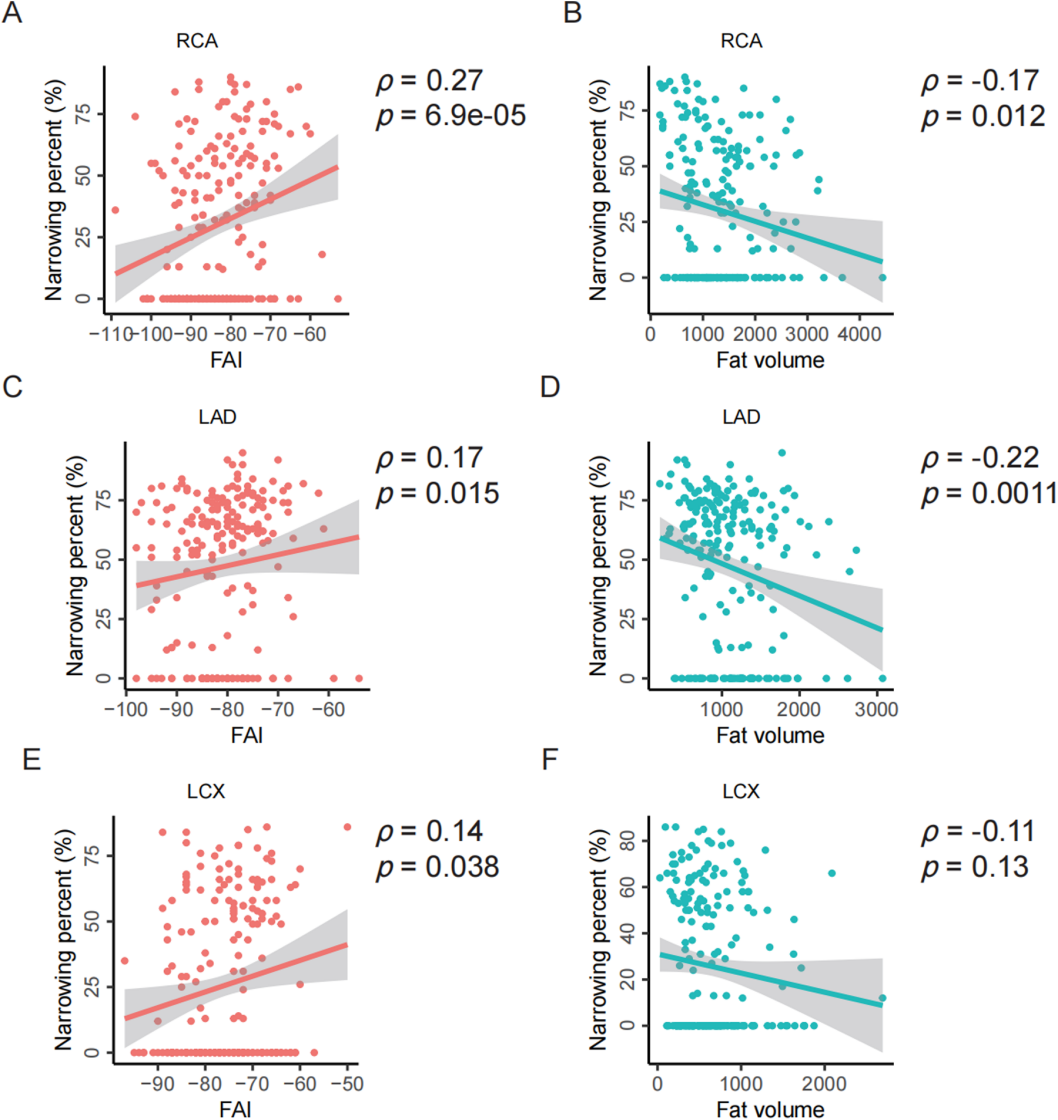
Spearman correlation analysis between PCAT parameters and stenosis of individual coronary arteries. **(A–B)** Correlation analysis between the FAI or fat volume and the stenosis of RCA. **(C–D)** Correlation analysis between the FAI or fat volume and the stenosis of LAD. **(E–F)** Correlation analysis between the FAI or fat volume and the stenosis of LCX. FAI, fat attenuation index; RCA, right coronary artery; LAD, left anterior descending artery; LCX, left circumflex artery; *ρ*, Spearman correlation, *p*, *p*-value.

For the RCA, the baseline characteristics are presented in Table 3. Both univariate and multivariate analyses demonstrated a significant association between FAI and RCA stenosis. The multivariate analysis indicated that the FAI of the RCA had an independent effect on stenosis, with higher FAI values associated with an increased risk of RCA stenosis (OR: 1.041, 95% CI: 1.003 to 1.081). This association was further supported by a significant positive correlation between FAI and RCA stenosis (*ρ* = 0.27, *p* = 6.9e-05; Figure 5A). Additionally, a weak but significant negative correlation was observed between fat volume and RCA stenosis (*ρ* = −0.17, *p* = 0.012; Figure 5B).

**Table 3:** Comparison of the characteristics with and without stenosis of RCA.

Variables	RCA narrowing		Univariate		Multivariate	
	No (*n* = 87)	Yes (*n* = 120)	*t*/*z*/*χ^2^*	*p*-value	*β*	*p*-value
Age (y)	57.38 ± 10.50	61.72 ± 10.54	2.938	0.004*	0.050	0.004*
BMI (kg/m^2^)	25.53 ± 3.27	25.69 ± 3.62	0.315	0.753		
Gender (M/F)	43/45	77/42	5.211	0.022*	−0.694	0.064
Smoking (N/Y)	68/20	79/40	2.913	0.088		
Drinking (N/Y)	74/14	86/33	4.029	0.045*	0.643	0.155
Hypertension (N/Y)	55/33	48/71	9.941	0.002*	0.538	0.122
Diabetes (N/Y)	81/7	77/42	20.928	<0.001*	1.517	0.006*
Dyslipidemia (N/Y)	40/48	49/70	0.378	0.529		
Arrhythmia (N/Y)	66/22	85/34	0.327	0.567		
TG (mmol/L)	1.53 ± 0.76	2.02 ± 1.48	2.480	0.013*	0.405	0.041*
LDL-c (mmol/L)	2.84 ± 0.81	2.83 ± 0.86	0.141	0.888		
Cholesterol (mmol/L)	4.70 ± 1.07	4.51 ± 1.19	1.152	0.250		
WBC (×10^9^/L)	6.79 ± 2.24	7.22 ± 2.08	1.897	0.058		
Uric acid (μmol/L)	347.91 ± 97.30	349.76 ± 90.26	0.292	0.770		
Serum K ^+^ (mmol/L)	3.99 ± 0.33	4.04 ± 0.39	0.337	0.736		
FBG (mmol/L)	5.67 ± 1.31	6.54 ± 2.20	3.108	0.002*	0.018	0.887
BNP (ng/mL)						
≤100	75 (85.23%)	96 (80.67%)	0.731	0.393		
>100	13 (14.77%)	23 (19.33%)				
D-dimer (ng/mL)						
≤600	81 (92.05%)	97 (81.51%)	4.659	0.031*	0.670	0.210
>600	7 (7.95%)	22 (18.49%)				
Creatinine (μmol/L)						
≤97	83 (94.32%)	107 (89.92%)	1.301	0.254		
>97	5 (5.68%)	12 (10.08%)				
PCAT volume (RCA) (mm^3^)	1,389.82 ± 714.81	1,292.17 ± 712.45	1.008	0.313	−0.005	0.851
FAI (RCA) (HU)	−85.40 ± 9.11	−81.32 ± 9.57	3.092	0.002*	0.040	0.033*

BMI, body mass index; BNP, brain natriuretic peptide; FBG, fasting blood glucose; FAI, fat attenuation index; PCAT, pericoronary adipose tissue; RCA, right coronary artery; TG, triacylglycerol; WBC, white blood cell; M/F, male/female; N/Y, no/yes.

* *p* < 0.05.

For the LAD, similar analyses were conducted. The fat volume of the LAD was found to have an independent effect on stenosis, with higher fat volume associated with a lower risk of LAD stenosis (OR: 0.884, 95% CI: 0.809 to 0.965; Supplementary Table S1). Consistent with RCA, the FAI is significantly positively correlated with the stenosis of LAD (*ρ* = 0.17, *p* = 0.015; Figure 5C) while the fat volume is significantly negatively correlated with the stenosis of LAD (*ρ* = −0.22, *p* = 0.0011; Figure 5D).

For the LCX, neither fat volume nor FAI showed a significant independent effect on stenosis (Supplementary Table S2). However, consistent with RCA and LAD, the FAI is significantly positively correlated with the stenosis of LCX (*ρ* = 0.14, *p* = 0.038; Figure 5E) while the fat volume is insignificantly negatively correlated with the stenosis of LCX (*ρ* = −0.11, *p* = 0.13; Figure 5F).

These findings highlight the significant association between PCAT and artery stenosis, while also illustrating that the effects of PCAT on stenosis vary across different coronary arteries.

### 3.4 Association between PCAT and artery plaque characteristics

Plaque composition is a crucial feature in patients with CAD. To explore whether PCAT is significantly associated with plaque characteristics, we categorized CAD patients into three groups based on plaque type: calcified, non-calcified, and mixed plaques (see Methods). Two key PCAT parameters—FAI and PCAT volume—were compared across these plaque groups, with the results summarized in Table 4.

**Table 4:** Comparison of pericoronary adipose tissue metrics among different LAD, LCX, and RCA plaque properties in patients with coronary artery disease.

PCAT parameters	Non-calcified plaque	Calcified plaque	Mixed plaque	Statistics	*p*-value
LCX volume (mm^3^)	622.22 ± 441.27	447.59 ± 345.99	671.58 ± 391.53	*H* = 8.869	0.012*
LCX FAI (HU)	−75.16 ± 7.69	−73.84 ± 7.73	−74.93 ± 8.31	*F* = 0.209	0.811
PCAT parameters	Non-calcified plaque	Calcified plaque	Mixed plaque	Statistics	*p*-value
LAD volume (mm^3^)	1,100.20 ± 416.61	742.10 ± 548.22	1,024.42 ± 457.60	*H* = 5.859	0.053
LAD FAI (HU)	−78.84 ± 6.40	−81.00 ± 7.48	−81.09 ± 7.77	*F* = 1.276	0.282
PCAT parameters	Non-calcified plaque	Calcified plaque	Mixed plaque	Statistics	*p*-value
RCA volume (mm^3^)	1,330.35 ± 728.15	1,101.24 ± 601.12	1,253.64 ± 707.96	*H* = 1.416	0.493
RCA FAI (HU)	−82.19 ± 9.25	−79.64 ± 7.97	−81.63 ± 9.49	*F* = 0.368	0.693

FAI, fat attenuation index; LAD, left anterior descending; LCX, left circumflex artery; RCA, right coronary artery.

* *p* < 0.05.

PCAT volume: For the LCX, the mean PCAT volume was 622.22 ± 441.27 mm^3^ in the NCP group, 447.59 ± 345.99 mm^3^ in the CP group, and 671.58 ± 391.53 mm^3^ in the mixed plaque group. The differences in PCAT volume among the plaque types were statistically significant (Kruskal-Wallis H test, *H* = 8.869, *p* = 0.012). Similarly, although not statistically significant, the mean PCAT volume for CP in the LAD and RCA was lower than that for NCP and mixed plaques.

FAI values: For the LCX, no significant differences were observed in FAI values across the plaque groups (ANOVA, *F* = 0.209, *p* = 0.811), with mean FAI values of −75.16 ± 7.69 HU for NCP, −73.84 ± 7.73 HU for CP, and −74.93 ± 8.31 HU for mixed plaques. Similarly, no significant differences in FAI values were found for the LAD or RCA across the different plaque groups.

These findings suggest that PCAT volume is significantly associated with plaque composition in CAD patients, particularly in the LCX, although the associations vary by different arterial types. Combined with previous results, our analysis indicates that both FAI and PCAT volume are closely linked to CAD risk, arterial stenosis, and plaque characteristics. This highlights the potential diagnostic value of incorporating PCAT parameters into CCTA-based assessments. In the future, integrating quantitative PCAT metrics in CCTA evaluations for patients with suspected CAD may improve the comprehensive assessment of disease severity and associated risks.

### 3.5 PCAT parameters improving CCTA’s diagnostic performance

**Figure 6:**
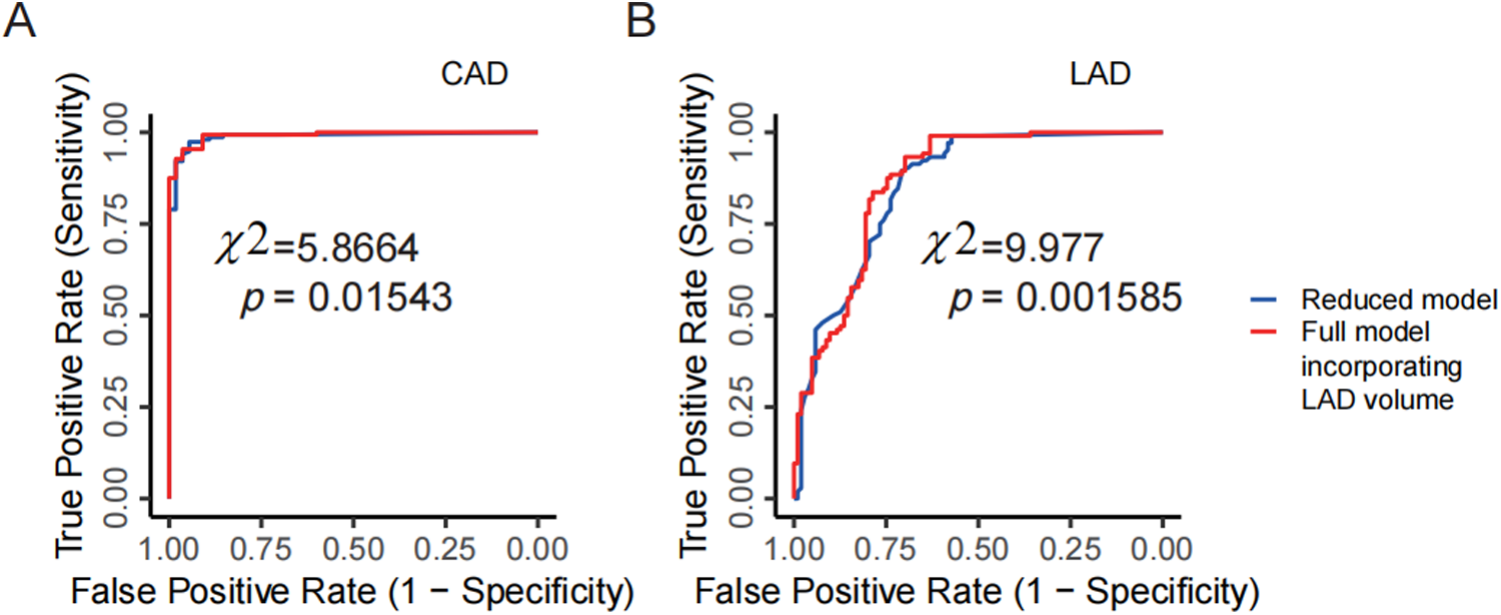
PCAT parameters improving CCTA's diagnostic performance. **(A)** Comparison between the full model and the reduced model for predicting CAD in DSA examinations. The reduced model includes the narrowing percentages of the three coronary arteries (LAD, LCX, RCA) from CCTA as predictors, while the full model incorporates the LAD volume as an additional predictor. **(B)** Comparison between the full model and the reduced model for predicting severe vs. non-severe stenosis of the LAD in DSA examinations. The reduced model uses the narrowing percentage of the LAD from CCTA, and the full model adds LAD volume as an additional predictor. CCTA, coronary computed tomography angiography; DSA, digital subtraction angiography; LAD, left anterior descending artery; LCX, left circumflex artery; RCA, right coronary artery.

Although this study has a moderate sample size, we sought to explore whether incorporating PCAT parameters into CCTA measurements of arterial narrowing could significantly improve CAD prediction. Using DSA-diagnosed CAD as the gold standard, we first built a multivariate logistic model that included the narrowing percentage of the three main coronary arteries (LAD, LCX, and RCA), along with the FAI and fat volume of these arteries, to predict CAD. Next, variables with *p* < 0.1 were selected to create a refined multivariate logistic model (the “full model”). For comparison, we also developed a “reduced model” that included only the narrowing percentages of the three arteries. An LRT based on the chi-square distribution was performed to determine whether the full model showed a significant improvement over the reduced model. The results showed that the narrowing percentages of all three arteries had a significant effect on CAD prediction (*p* = 0.00785 for LCX, *p* = 7.51e-06 for LAD, and *p* = 0.00477 for RCA). Moreover, the fat volume of the LAD had a significant independent effect in the full model (*p* = 0.03079). The LRT indicated that the full model significantly improved predictive performance compared to the reduced model (*p* = 0.01543; Figure 6A), suggesting that the fat volume of the LAD plays a significant role in predicting CAD.

Using the same analytical framework, we further analyzed each artery individually and still took the severe vs. non-severe stenosis determined by DSA as the gold standard. For example, in the case of the LAD, a multivariate logistic model was initially built to include the narrowing percentage, fat volume, and FAI to predict severe vs. non-severe stenosis. The narrowing percentage and the fat volume of the LAD with a *p*-value smaller than 0.1 were chosen to build the full model. In this full model, both the narrowing percentage and fat volume of the LAD were significant at the 0.05 level (*p* = 2.47e-12 for narrowing percentage, and *p* = 0.00514 for fat volume). A reduced model was then constructed using only the narrowing percentage of the LAD. Through the LRT, the full model demonstrated a significant improvement over the reduced model (*p* = 0.001585; Figure 6B), indicating that including the fat volume of the LAD significantly improves CCTA's predictive performance for the stenosis of the LAD. However, similar analyses for the RCA and LCX did not show significant improvements based on LRT results.

These findings demonstrate that incorporating PCAT parameters, particularly fat volume, can significantly enhance the diagnostic performance of CCTA for CAD, as well as for predicting the stenosis of LAD. In the future, incorporating PCAT parameters into CCTA assessments with a larger patient sample could help develop a more robust diagnostic model.

## 4 Discussion

This study emphasizes the potential of PCAT as a crucial factor in enhancing the diagnostic accuracy of CCTA for predicting CAD. Given PCAT's pivotal role in CAD pathogenesis, establishing reliable PCAT-based markers is essential to improve CCTA's diagnostic performance. These markers can enable more accurate identification of CAD, facilitating the development of a more personalized diagnostic protocol for patients with suspected CAD. By increasing diagnostic precision, this approach allows for more tailored patient assessments and more effective management of individual risk profiles. Additionally, this non-invasive imaging method offers significant benefits for patients who are hesitant to undergo invasive procedures like DSA. It can also help reduce the need for fractional flow reserve (FFR) measurements, and avoid extra imaging protocols or increased radiation exposure (21).

PCAT has emerged as a critical player in the pathogenesis of CAD due to its strong association with vascular inflammation. CAD is now widely recognized as a chronic vascular inflammatory condition, with substantial evidence—from histological studies to clinical trials—highlighting the role of inflammation in the development of atherosclerotic plaques and the occurrence of major adverse cardiac events (30). However, conventional systemic biomarkers fall short in localizing coronary inflammation, leading to the exploration of non-invasive alternatives like CCTA for assessing inflammatory risk. *in vitro* studies have demonstrated that pro-inflammatory molecules released from the inflamed vessel wall disrupt the differentiation and lipid accumulation of PCAT preadipocytes, resulting in smaller adipocytes (31, 32). This inflammatory response is mediated by the release of cytokines and vasoactive mediators such as TNF-α, IL-6, IL-7, and angiotensin through vascular and paracrine signaling pathways. This cascade exacerbates coronary endothelial dysfunction and induces lipid profile alterations that accelerate atherosclerosis progression (33–35). These findings highlight the importance of standardized PCAT quantification and consistent scanning parameters when evaluating PCAT in clinical practice.

Our findings demonstrate that CT attenuation of PCAT, measured as the FAI, is a valuable marker for assessing both the CAD and coronary artery stenosis. We observed significantly higher mean FAI values in the proximal coronary arteries of the CAD group compared to the N-CAD group, with these differences being statistically significant. PCAT attenuation, as captured through CCTA, offers an indirect yet effective measure of coronary artery inflammation. Attenuation levels are highest near the vessel wall and decrease with increasing radial distance, forming a characteristic attenuation gradient. Notably, this gradient is steeper in inflamed PCAT compared to normal PCAT (5), which enhances CCTA's diagnostic capability for identifying functionally significant coronary stenosis. Recent research supports these findings, showing that increased CT attenuation of PCAT around the RCA predicts major adverse cardiac events and plaque progression (36, 37), aligning with our results. The growing body of evidence underscores the feasibility of using PCAT attenuation to assess CAD and monitors inflammatory responses to therapies such as drug treatments or percutaneous coronary interventions. Although standardized CT thresholds for inflammation have yet to be established, non-invasive PCAT assessment shows promise for early detection of coronary inflammation, improving preventive strategies for atherosclerosis (38).

The study also found that PCAT fat volume is a valuable marker for assessing the CAD and coronary artery stenosis. The PCAT volume was significantly lower in the CAD group, likely due to perivascular attenuation shifting the PCAT towards the aqueous phase in these patients. Similarly, Balcer et al. reported a strong independent correlation between PCAT volume and culprit lesions in patients with acute coronary syndrome (ACS) (39). However, unlike our findings, their study associated higher PCAT volume with an increased risk of acute myocardial infarction (AMI). This discrepancy may be due to our focus on proximal vessels or differences in patient populations and imaging modalities. We chose the proximal vessels followed the previous many studies (31, 37, 40). Furthermore, the CAD group in our study exhibited a higher prevalence of adipose precursor cells with reduced fat content, adding complexity to the interpretation of PCAT volume. Further analysis, such as pathological assessments of PCAT cell morphology, is needed to clarify these findings (14, 20).

The sensitivity of CCTA for diagnosing RCA stenosis is often lower than for the LAD and LCX, and several factors likely contribute to this disparity. First, the RCA typically has a smaller diameter than the LAD and LCX and supplies a smaller portion of the heart. As a result, stenosis in the RCA may cause less pronounced ischemia, making it harder to detect through non-invasive imaging like CCTA (22). Additionally, the anatomical position and angle of the RCA within the chest increase its susceptibility to imaging artifacts during non-invasive procedures, further diminishing CCTA's sensitivity for detecting RCA stenosis. In contrast, the LAD and LCX are generally easier to visualize, contributing to higher sensitivity in detecting stenosis in these arteries (41, 42).

Our findings indicate that the FAI of the RCA has a significant independent effect on RCA stenosis but not on the LAD or LCX, whereas the fat volume of the LAD shows a significant independent effect on LAD stenosis but not on the RCA or LCX. First, these variable associations between FAI or fat volume and the stenosis across the RCA, LAD, and LCX may stem from anatomical differences (36). For instance, the RCA has fewer side branches, more surrounding adipose tissue, and a more consistent lumen diameter, while the lumen of the LAD and LCX progressively narrows from their origin to the periphery (36). Second, previous studies have demonstrated that PCAT attenuation around the proximal RCA is a highly standardized and reproducible method for evaluating CAD across diverse patient cohorts (31, 37, 40). Goeller et al., for example, found that changes in PCAT attenuation around the proximal RCA correlated with variations in non-calcified plaque burden throughout the coronary tree (37). Likewise, Antonopoulos et al. showed that PCAT attenuation was higher around culprit lesions in patients with acute coronary syndrome (ACS) compared to non-culprit or stable lesions (40). These findings suggest that while the FAI of RCA has a clear independent effect on RCA stenosis, its lack of effect on the LCX and LAD requires further investigation. These also could reflect the complexity of CAD, where each artery's environment and risk factors are distinct. Nevertheless, the consistent correlations across different coronary arteries point to potentially related underlying mechanisms.

The relationship between FAI values or fat volume and the stenosis of the three coronary arteries, though showing only weak correlations, should not be dismissed as artificial bias. First, the direction of the correlation is consistent across different coronary arteries, lending robustness to these findings. Second, statistical significance is typically assessed with a *p*-value threshold of 0.05, meaning that there is less than a 5% chance that the observed relationship occurred by random chance (43). This widely accepted benchmark suggests that even weak correlations can be meaningful when the *p*-value is below this threshold. Third, in complex conditions like CAD, multiple factors contribute to disease presentation, so individual metrics rarely show large effects. Nevertheless, our multivariate logistic regression analysis confirmed that some PCAT parameters have a significant independent effect on stenosis in individual arteries, which can provide valuable insights into disease mechanisms and may help isolate more relevant pathophysiological aspects. Lastly, the weak correlations observed in this study could largely be attributed to the small sample size (27), a limitation that future studies with larger clinical cohorts could address.

Our study found that the fat volume of CP remains minimal compared to NCP and mixed plaques, with this difference being statistically more significant in the LCX. This is likely due to greater variability in LCX alignment and individual anatomical differences compared to the RCA and LAD. Such variability underscores the need for a larger sample size to further investigate the relationship between PCAT characteristics and plaque composition across different coronary arteries. Zhan et al. previously demonstrated that individuals with diabetes, hypertension, elevated inflammatory markers, and high-risk plaques are more prone to developing coronary plaques, particularly high-risk ones (44). Consistent with these findings, increased PCAT attenuation has been associated with a higher risk of cardiac death, independent of the presence of high-risk plaques (15). These observations emphasize the importance of understanding the complex interplay between PCAT properties and plaque composition in CAD patients, especially as it varies between different coronary arteries.

This research leveraged AI technology to accurately and efficiently segment and quantify PCAT images, showcasing its potential in cardiovascular disease diagnosis and risk prediction. The use of AI in cardiac imaging is gaining traction, offering promising opportunities to expand the diagnostic value of cardiac adipose tissue analysis. By employing machine learning, more detailed insights can be extracted from PCAT metrics, which play a crucial role in diagnosing and predicting CAD (45). For patients undergoing CCTA, particularly those with borderline stenosis (around 50%), integrating PCAT attenuation and volume measurements may provide valuable guidance on whether further invasive procedures, such as DSA, are warranted. This combined approach could significantly enhance both patient assessment and clinical management, allowing for more tailored and precise treatment strategies.

## 5 Limitations

Our research has several limitations. First, being a cross-sectional analysis with retrospective data collection, there is potential for selection bias. Second, the relatively small sample size may limit the generalizability of the results. Larger-scale prospective studies are needed to validate this. Third, some baseline data were collected through patients recall, which could introduce recall bias. Fourth, the decision to perform percutaneous coronary intervention was made at the discretion of the operator, potentially introducing treatment selection bias. Lastly, PCAT attenuation measured by CCTA serves as a surrogate marker for coronary artery inflammation, relying on average voxel intensity values without accounting for spatial relationships between voxels. However, our data were processed using AI-based methods, which help exclude known confounding factors that may affect the CT-derived attenuation values. Furthermore, the consistency across different analyses and coronary arteries supports the robustness of our findings.

## 6 Conclusion

The study demonstrates that incorporating PCAT metrics, particularly the FAI and fat volume, significantly enhances the diagnostic performance of CCTA for assessing CAD and arterial stenosis, which further emphasizes the importance of adhering to standardized PCAT quantification methods. CCTA showed high overall diagnostic accuracy for CAD, but its performance varied across different arteries, with the LAD showing the highest sensitivity. The FAI of RCA was independently associated with an increased risk of CAD, while higher PCAT volume was associated with a reduced risk of CAD. In particular, FAI significantly predicted stenosis in the RCA, while fat volume was inversely correlated with stenosis in the LAD. PCAT volume was also significantly associated with plaque characteristics, with lower volumes observed in CP. Overall, PCAT has potential application value in the comprehensive assessment of CAD. Future studies are necessary to validate the validity of PCAT as a prognostic marker and to assess whether PCAT-targeted therapy improves the prognosis of patients with CAD.

## Data Availability

The raw data supporting the conclusions of this article will be made available by the authors, without undue reservation.
