# Corrigendum: Evaluating the role of pericoronary adipose tissue on coronary artery disease: insights from CCTA on risk assessment, vascular stenosis, and plaque characteristics

**DOI:** 10.3389/fcvm.2024.1531099

**Published:** 2024-11-28

**Authors:** Jingyue Wang, Huicong Zhang, Zihao Wang, Wenyun Liu, Dianbo Cao, Qian Tong

**Affiliations:** ^1^ Department of Cardiovascular Medicine, The First Hospital of Jilin University, Changchun, China; ^2^ Department of Radiology, The First Hospital of Jilin University, Changchun, China

**Keywords:** pericoronary adipose tissue (PCAT), coronary artery disease (CAD), coronary computed tomography angiography (CCTA), digital subtraction angiography (DSA), fat attenuation index (FAI)

A Corrigendum on Evaluating the role of pericoronary adipose tissue on coronary artery disease: insights from CCTA on risk assessment, vascular stenosis, and plaque characteristics By Wang J, Zhang H, Wang Z, Liu W, Cao D and Tong Q (2024). Front. Cardiovasc. Med. 11:1451807. doi: 10.3389/fcvm.2024.1451807

In the published article, there was an error in the Funding statement. The grant number for the “National Key R&D Program of China” was displayed as “2022YFA0806400”. The correct funding statement appears below.

