# Supplementary material for: Evaluating the role of pericoronary adipose tissue on coronary artery disease: insights from CCTA on risk assessment, vascular stenosis, and plaque characteristics
Source: Front Cardiovasc Med. 2024 Oct 23;11:1451807. doi: 10.3389/fcvm.2024.1451807 (PMC11538997; doi:10.3389/fcvm.2024.1451807)
Supplement: Supplementary file 1 [file Table1.docx]

**Supplementary Table 1** Comparison of the characteristics with and without stenosis of LAD.

| **Variables** | **LAD narrowing** | | **Univariate** | | **Multivariate** | |
| --- | --- | --- | --- | --- | --- | --- |
|  | **No (*n*=52)** | **Yes (*n*=155)** | ***t*/z/*χ^2^*** | ***p-*value** | ***β*** | ***p-*value** |
| Age (y) | 55.81 ± 10.33 | 61.24 ± 10.53 | 3.234 | 0.001* | 0.054 | 0.012* |
| BMI (kg/m^2^) | 24.80 ± 3.36 | 25.90 ± 3.47 | 1.997 | 0.047* | 0.167 | 0.019* |
| Gender (M/F) | 26/26 | 94/61 | 1.811 | 0.178 |  | |
| Smoking (N/Y) | 39/13 | 108/47 | 0.536 | 0.464 |  | |
| Drinking (N/Y) | 40/12 | 120/35 | 0.005 | 0.941 |  | |
| Hypertension (N/Y) | 40/12 | 63/92 | 20.498 | <0.001* | 1.228 | 0.004* |
| Diabetes (N/Y) | 47/5 | 111/44 | 7.594 | 0.006* | 0.202 | 0.730 |
| Dyslipidemia (N/Y) | 28/24 | 61/94 | 3.336 | 0.068 |  | |
| Arrhythmia（N/Y） | 42/10 | 109/46 | 2.153 | 0.142 |  | |
| TG (mmol/L) | 1.41 ± 0.75 | 1.95 ± 1.35 | 3.335 | <0.001* | 0.516 | 0.041* |
| LDL-c (mmol/L) | 2.70 ± 0.74 | 2.88 ± 0.87 | 1.290 | 0.198 |  | |
| Cholesterol (mmol/L) | 4.54 ± 0.84 | 4.61 ± 1.23 | 0.420 | 0.675 |  | |
| WBC (×10ˆ9/L) | 6.36 ± 2.08 | 7.26 ± 2.14 | 3.182 | 0.001* | 0.202 | 0.066 |
| Uric acid (μmol/L) | 337.85 ± 101.68 | 352.71 ± 90.07 | 1.411 | 0.158 |  | |
| Serum K^+^(mmol/L) | 4.04 ± 0.27 | 4.01 ± 0.39 | 0.600 | 0.549 |  | |
| FBG (mmol/L) | 5.49 ± 1.10 | 6.40 ± 2.08 | 3.115 | 0.002* |  | |
| BNP (ng/mL) |  |  |  |  |  |  |
| ≤100 | 49 (94.23%) | 122 (78.71%) | 6.529 | 0.011* | 1.376 | 0.067 |
| >100 | 3 (5.77%) | 33 (21.29%) |  |  |  |  |
| D-dimer (ng/mL) |  |  |  |  |  |  |
| ≤600 | 51 (98.08%) | 127 (81.94%) | 8.421 | 0.004* | 2.726 | 0.017* |
| >600 | 1 (1.92%) | 28 (18.06%) |  |  |  |  |
| Creatinine (μmol/L) |  |  |  |  |  |  |
| ≤97 | 48 (92.31%) | 142 (91.61%) | 0.000 | 1.000 |  | |
| >97 | 4 (7.69%) | 13 (8.39%) |  |  |  | |
| PCAT volume (LAD) (mm^3^) (mm³) | 1,234.51 ± 538.00 | 1,056.14 ± 469.12 | 2.221 | 0.026* | 0.123 | 0.006* |
| FAI (LAD) (HU) | -81.40 ± 7.80 | -80.88 ± 7.64 | 0.988 | 0.323 | 0.032 | 0.270 |

BMI, body mass index; BNP, brain natriuretic peptide; FBG, fasting blood glucose; FAI, fat attenuation index; LAD, left anterior descending artery; PCAT, pericoronary adipose tissue; TG, triacylglycerol; WBC, white blood cell. M/F, male/female; N/Y, no/yes.

* *p* < 0.05.
