# Supplementary material for: Evaluating the role of pericoronary adipose tissue on coronary artery disease: insights from CCTA on risk assessment, vascular stenosis, and plaque characteristics
Source: Front Cardiovasc Med. 2024 Oct 23;11:1451807. doi: 10.3389/fcvm.2024.1451807 (PMC11538997; doi:10.3389/fcvm.2024.1451807)
Supplement: Supplementary file 2 [file Table2.docx]

**Supplementary Table 2** Comparison of the characteristics with and without stenosis of LCX.

| **Variables** | **LCX narrowing** | | **Univariate** | | **Multivariate** | |
| --- | --- | --- | --- | --- | --- | --- |
|  | **No (*n*=108)** | **Yes (*n*=99)** | ***t/z*/*χ^2^*** | ***p-*value** | ***β*** | ***p-*value** |
| Age (y) | 57.43 ± 10.54 | 62.55 ± 10.31 | 3.527 | ＜0.001* | 0.060 | 0.001* |
| BMI (kg/m^2^) | 25.08 ± 3.28 | 26.22 ± 3.59 | 2.377 | 0.018* | 0.172 | 0.001* |
| Gender (M/F)  Male | 60/40 | 60/39 | 0.541 | 0.462 |  | |
| Smoking (N/Y) | 76/32 | 71/28 | 0.046 | 0.831 |  | |
| Drinking (N/Y) | 85/23 | 75/24 | 0.255 | 0.613 |  | |
| Hypertension (N/Y) | 64/44 | 39/60 | 8.154 | 0.004* | 0.505 | 0.119 |
| Diabetes (N/Y) | 92/16 | 66/33 | 9.804 | 0.002* | 0.990 | 0.029* |
| Dyslipidemia (N/Y) | 48/60 | 41/58 | 0.194 | 0.660 |  |  |
| Arrhythmia (N/Y) | 81/27 | 70/29 | 0.482 | 0.487 |  | |
| TG (mmol/L) | 1.79 ± 1.41 | 1.84 ± 1.06 | 1.358 | 0.175 |  | |
| LDL-c (mmol/L) | 2.85 ± 0.76 | 2.82 ± 0.92 | 0.293 | 0.770 |  | |
| Cholesterol (mmol/L) | 4.62 ± 0.98 | 4.57 ± 1.30 | 0.246 | 0.806 |  | |
| WBC (×10ˆ9/L) | 6.81 ± 2.10 | 7.28±2.19 | 1.697 | 0.090 |  | |
| Uric acid (μmol/L) | 346.01 ± 99.72 | 352.21 ± 85.66 | 0.478 | 0.633 |  | |
| Serum K^+^(mmol/L) | 4.01 ± 0.29 | 4.02 ± 0.43 | 0.686 | 0.492 |  | |
| FBG (mmol/L) | 6.02 ± 2.00 | 6.34 ± 1.82 | 2.131 | 0.033* | -0.126 | 0.218 |
| BNP (ng/mL) |  | | | | | |
| ≤100 | 93 (86.11%) | 78 (78.79%) | 1.928 | 0.165 |  | |
| ＞100 | 15 (13.89%) | 21 (21.21%) |  |  |  | |
| D-dimer (ng/mL) |  | | | | | |
| ≤600 | 99 (91.67%) | 79 (79.80%) | 6.040 | 0.014* | 0.855 | 0.078 |
| ＞600 | 9 (8.33%) | 20 (20.20%) |  |  |  |  |
| Creatinine (μmol/L) |  | | | | | |
| ≤97 | 100 (92.59%) | 90 (90.91%) | 0.194 | 0.659 |  | |
| ＞97 | 8 (7.41%) | 9 (9.09%) |  |  |  | |
| PCAT volume (LCX) (mm^3^) (mm³) | 667.41 ± 393.98 | 646.59 ± 438.60 | 0.782 | 0.434 | -0.025 | 0.552 |
| FAI (LCX) (HU) | -76.69 ± 8.22 | -75.09 ± 8.04 | 1.409 | 0.160 | 0.024 | 0.284 |

BMI, body mass index; BNP, brain natriuretic peptide; FBG, fasting blood glucose; FAI, fat attenuation index; LCX, left circumflex artery; PCAT, pericoronary adipose tissue; TG, triacylglycerol; WBC, white blood cell; M/F, male/female; N/Y, no/yes.

**p* < 0.05.
